# Who gets what, where: To what extent are inequalities in distribution of Norwegian child welfare service measures related to spatial and temporal variation

**DOI:** 10.1371/journal.pone.0336104

**Published:** 2025-11-03

**Authors:** Norunn Hornset

**Affiliations:** Department of Geography, Norwegian University of Science and Technology, Trondheim, Norway; Universidad de Chile, CHILE

## Abstract

Inequality has long been a persistent issue, but it has intensified since the turn of the millennium and is now considered one of the most urgent challenges of our time. Even in Norway, which is traditionally considered an egalitarian society, there is an increase in income inequalities. Inequality disproportionately affects marginalized groups; one especially marginalized group is young people with experience in child welfare services. Traditionally, inequality in child welfare services has been explored from individual or family perspectives. However, it has been argued that spatial and temporal dimensions also influence the distribution of these services. This paper looks beyond individual and family factors by examining how temporal and spatial factors affect distribution of child welfare services in Norway. Using registry data for individuals born in Norway in 1995 and 2005, the study combines logistic regression analysis with hotspot analysis (Getis-Ord Gi*) in GIS. The findings reveal that both spatial and temporal factors significantly influence the distribution of child welfare services in Norway. In particular, living in rural municipalities is associated with reduced access to services. As a result, the national principle of equal access to services across all regions is not being upheld.

## 1. Introduction

Since the turn of the millennium, global inequality has been on the rise and is now recognized as a major societal challenge [1–3]. Although Norway is often regarded as an egalitarian society, income inequality has increased, and the share of children living in persistent poverty has grown from 4% to 11% over the past three decades [4]. These disparities increase the risk of disadvantage for vulnerable groups, and one of the most vulnerable groups in Norway is young people with experience from child welfare services [5,6]. Child welfare services aim to mitigate such risks, yet research shows that inequalities persist. While most studies on child welfare services focus on individual and family characteristics, scholars argue that including spatial and temporal perspectives is important for understanding how these inequalities emerge and persist [7–10]. This is particularly relevant in Norway, given the country’s administrative structure, which includes a large number of small municipalities tasked with delivering equal-quality public services regardless of their size or resource capacity [11].

Despite these concerns, empirical evidence on spatial and temporal inequalities in Norwegian child welfare services remains limited. This paper addresses this gap by examining how spatial and temporal factors influence the distribution of child welfare services in Norway. This study utilizes registry data for individuals born in Norway in 1995 and 2005, comprising 116,940 individuals. A four-step analytical approach was applied, combining logistic regression and hotspot analysis (Getis-Ord Gi*) in GIS. This approach tests whether individual and family characteristics alone can explain inequalities in child welfare services by [1] running a logistic regression model to explore how individual and family-level characteristics affect child welfare measures, [2] identifying spatial clustering through hotspot analysis, [3] expanding models with spatial variables, and [4] re-examining residual patterns.

The analysis reveals significant spatial clustering of child welfare measures, even after controlling for individual and family characteristics, and shows that these patterns vary across age groups. Results indicate that rural municipalities are associated with reduced access to services, while urban areas show hotspots of child welfare measures. Further, results emphasize temporal differences as the youngest age groups are significantly more likely to receive measures, compared to the older age groups. By including spatial and temporal perspectives, this study contributes to a more nuanced understanding of inequalities in child welfare services, highlighting the role of place and time beyond individual-level explanations.

## 2. Spatial and temporal inequality

Understanding inequality in child welfare services requires a multidimensional approach, where not only individual and family characteristics are considered, but also the spatial and temporal context in which these individuals live. Spatial inequality refers to how and why valued resources, such as public services, infrastructure, and opportunities, vary across places, and how these places themselves become both markers and makers of inequality [12]. Such a multidimensional understanding of inequality builds on a long-standing tradition within human geography of examining justice and welfare, drawing on foundational contributions from key thinkers such as David Harvey and Doreen Massey, who emphasize that spatial dimensions contribute to shaping patterns of inequality [10,13].

Research suggests that spatial inequality is shaped by three interrelated factors: economic structures, institutional arrangements, and geographic context [12]. In this paper, these dimensions will be referred to as ‘spatial characteristics’. Economic structures, such as available industries and labor opportunities, shape the accumulation of resources in an area based on the prestige and income levels related to available industries [10,14]. Such economic structures are tied to spatial characteristics. For instance, in rural areas, industries are often linked to agriculture and traditional labor, which typically offer lower wages and require less formal education, contributing to persistent patterns of disadvantage [15].

Institutional arrangements, such as the distribution of social benefits and the quality of public services, also play a critical role in reinforcing or limiting patterns of inequality [12]. Areas with favorable environments for productive activities tend to develop stronger institutional arrangements due to higher tax income and investments in physical and social infrastructure, attracting more capital and labor which reproduces patterns of affluence [10]. Other areas have lower institutional capacity and this disadvantage tend to accumulate, leading to lower quality and capacity of institutions due to limited resources and challenges in attracting and retaining a qualified workforce. Such challenges tend to be more present in rural areas [16–18].

Finally, geographic context, including population size, remoteness and physical characteristics, amplify or mitigate these inequalities [12]. While technological advances have reduced the need for proximity to resources, social structures continue to reproduce spatial patterns of affluence and deprivation [19].

While spatial perspectives reveal how inequality is distributed across places, a temporal lens highlights how these inequalities evolve and accumulate over time. Longitudinal approaches are important for understanding how life trajectories are shaped by both early experiences and ongoing interactions with institutions and environments [20,21]. Such patterns are reproduced across generations through family networks and neighborhood effects [9,22].

Temporal perspectives emphasize that individuals are embedded in social contexts that shift over time and are shaped by historical, relational, and institutional dynamics. For marginalized groups, early life events often serve as either risk or protective factors, with potential long-term implications for development and well-being [23]. Well-being itself is a multidimensional concept including health, happiness, security and quality of life [9,24] which is inherently spatial – people are well somewhere – but also temporal, as it reflects the unfolding of experiences over time. The availability of material, social, and psychological resources in a given place influences well-being, and these resources are often unevenly distributed both across regions and across the life course [25,26].

By synthesizing these perspectives, this study aims to explore how spatial and temporal dimensions shape the distribution of child welfare services in Norway. Recognizing that places are not neutral backdrops but active participants in the production of inequality, this approach allows for a more nuanced understanding of how disadvantage is produced, maintained, and potentially mitigated through policy and practice.

### 2.1 Inequality in child welfare services

Marginalized groups are often more exposed to the impacts of inequality [27]. One of the most vulnerable groups in society is young people with experience from child welfare services [5]. These individuals often face challenges related to physical and mental health, educational achievement, economic stability, and employment [28]. Young people with experience from child welfare services are more dependent on support from public services than their peers, making them more vulnerable to spatial inequality. In this context, inequality in child welfare services refers to systematic differences based on affluence and deprivation, which affect outcomes, chances, and experiences [7].

Research has tied these inequalities to geographical patterns, particularly levels of deprivation. In the UK, Bywaters and colleagues [7] have demonstrated how deprivation levels influence child welfare intervention rates, and describe findings supporting ‘the inverse intervention law’ where chances of receiving services are greater in affluent areas [7]. Similar findings have emerged in Norway, where Kojan and Storhaug [29] observed that more deprived municipalities tend to have lower intervention rates. Their findings also indicate that service provision is generally lower in rural areas with smaller municipal structures. These patterns suggest that where a child lives can significantly affect the support they receive, reinforcing the need to consider spatial dimensions in child welfare research.

Understanding these dynamics requires attention to the broader Norwegian context. Norway, has a small population relative to its total area. In 2017, it was divided into 426 municipalities, with half having populations of fewer than 5,000 people. The country’s commitment to maintaining existing settlement patterns is reflected in the generalist authority system, which requires all municipalities to maintain the same tasks, services, and development responsibilities regardless of local characteristics such as economy, population size, and settlement structures [11,30]. However, demographic shifts, particularly an aging and declining populations in smaller municipalities, pose significant challenges to this principle. These areas often struggle to attract and retain a competent workforce, which in turn affects the quality and consistency of public services, including child welfare [31]. The issue of small municipalities is a significant challenge in Norway today, leading to ongoing political discussions about merging municipalities.

In this context, municipalities are often forced to make difficult decisions about how to allocate limited resources. When prioritizing services, there is a tendency to focus on the youngest children, who are considered the most vulnerable [5,32,33]. This can lead to fewer available resources for young people who are transitioning out of care. One measure aimed at mitigating long-term negative outcomes for this group is the continuation of support after the age of 18, known as aftercare services. This support is intended to provide a more gradual and supported transition into adulthood, offering economic, practical, and emotional assistance [5,32]. Despite evidence linking aftercare to positive outcomes, access to such services may differ across municipalities, especially in municipalities with fewer people and resources, thus reinforcing spatial inequality in service provision.

## 3. Methods

### 3.1 Data

The data used in this study are based on a central public population registry, providing high-quality population data on Norwegian child welfare services [34]. This study utilizes yearly individual-level data from the complete 1995 and 2005 cohorts and their parents, spanning from 1995 to 2017 (n = 116,940). The use of panel data is advantageous, as it minimizes selection bias, nonresponse, and dropout-related challenges [35]. Additionally, data on Norwegian child welfare services are combined with yearly data from Norwegian municipalities to provide information on the geographical context. These municipal data are based on annual reports to the central government (KOSTRA) [36]. All data used in this study are obtained and provided by Statistics Norway.

### 3.2 Variable description

#### 3.2.1 Individual-level variables.

The outcome variable measures whether the young person had an active measure from child welfare services in a given year, including in-home care and out-of-home care. This is a binary variable, where 1 represents having an active measure and 0 represents not having an active measure from child welfare services in the given year. Following the arguments of Paulsen et al. [37] and Kääriälä [38], additional variables include gender, age, country background, and residential instability, as these are recognized as influential factors in the distribution of child welfare service measures. Residential instability is measured with two variables: the frequency of moves within a municipality and the frequency of moves between municipalities [39].

#### 3.2.2 Family-level variables.

Three variables are included to measure socioeconomic status in the family: household income, parents’ occupational prestige, and parental education. Household income represents the total yearly income, including social support for both parents, and is log-transformed to limit the impact of the skewed income distribution. Occupational prestige follows the Norwegian version of the International Standard Classification of Occupations (ISCO-08), with unemployed individuals included in the lowest category [40]. The education variable represents the highest achieved level of education based on the Norwegian Classification of Education [41].

#### 3.2.3 Municipality-level variables.

Spatial inequality is a central aspect of this paper and is shaped by the interplay between economic structures, institutional arrangements, and geographic context [12]. However, it is worth noting that although variables are used to describe different aspects of spatial inequality, it is not possible to set clear boundaries between the different factors, as they influence and are influenced by each other.

Degree of urbanization captures the geographic context of municipalities, reflecting differences in population size, remoteness, and settlement structures. Based on the Norwegian adaptation of Eurostat’s Degree of Urbanization Classification scale [42,43], municipalities are categorized on a scale from 1 (urban) to 3 (rural). This classification aligns with arguments that rural areas often face disadvantage due to physical distance, smaller populations, and limited resources [12,19]. Unemployment rates reflect economic structures and represent the availability and quality of the local labor market. As Harvey [10] and Massey [14] argue, economic structures shape the accumulation of resources and opportunities in a given area. High unemployment can contribute to reduced investments in the local area, thus lowering public investment, and is therefore often used as a representation of deprivation in an area [44,45]. Unemployment is measured as the percentage of individuals registered as unemployed in the municipality, with data provided by Statistics Norway [46]. The number of public housing units per 1,000 inhabitants reflects institutional arrangements, specifically, the capacity of municipalities to provide social infrastructure. Note that this variable lacks information for the first six years of the time series and is thus not included in model 1.5. Finally, life expectancy reflects both economic and institutional conditions as it is known to be affected by long-term determinants such as health, income, education, living conditions, and the local environment. Life expectancy is measured in years for each municipality and year, with data collected by the Norwegian Institute of Public Health [47].

Together, these variables reflect the argument made by Hooks et al., [12] that spatial inequality is produced and maintained through structural, institutional, and geographic mechanisms.

### 3.3 Research design

This study employs a four-stage research design, following the approach of Halvorsen et al. [48]. The first stage involves a logistic regression model to explore how individual and family-level variables affect the provision of child welfare service measures. Given the panel structure of the data, yearly dummies are included to control for repeated observations over time. Furthermore, all models are performed with cluster-correlated standard errors, which control for repeated observations of the same individuals. This approach was chosen rather than a multilevel approach, as multilevel models should be based on successive sampling. Since the structures in this dataset are not stable over time, this assumption is violated [49]. A significant number of the young people represented in the dataset move between municipalities one or several times during the time series, further violating this assumption. Fixed effects for municipalities were considered but ultimately rejected, as this more conservative approach would reduce the effect of municipality characteristics (by design). Additionally, a fixed effects approach would decrease the model’s degree of freedom. Consequently, municipality fixed effects would be less efficient use of the data [50]. This model is run for four different age groups, based on findings by Drange et al. [51] that the provision of services varies with age. The age groups are 1) 0–5 years, 2) 6–12 years, 3) 13–18 years, and 4) 19–22 years. The purpose of this model is to calculate deviation from the estimate, assuming that not all variation in child welfare service provision is captured by the error term.

The key rationale of the paper is that individual and family characteristics cannot fully explain patterns of inequality. This assumption is tested in the second stage, which involves exploring the spatial distribution of residuals to identify potential spatial clustering. Non-random distribution of residuals suggests that underlying, non-random factors are causing these patterns. The high-value patterns are of particular interest, as they indicate the lowest agreement with the original model. Clusters are identified using the Getis-Ord Gi* statistics in GIS, commonly referred to as hotspot analysis [52]. This method reports z-scores, p-values, and Gi_Bin values to assess spatial clustering. A cluster is classified as a hotspot if it has a positive Gi_Bin value and a p-value below the conventional threshold of 0.05; these are visualized in shades of red on the map, with color intensity reflecting the level of statistical significance. Conversely, coldspots are characterized by negative Gi_Bin values and similarly significant p-values, and are displayed in shades of blue. Areas with Gi_Bin values of 0 indicate no statistically significant clustering and are shown in white [52]. If the hotspot analysis reveals significant spatial clustering, this suggests that the model does not fully account for spatial variation, indicating the presence of unexplained variance. To reduce this, the model should be extended to include additional theoretically grounded predictors. Drawing on the literature review in this paper, incorporating spatial variables is a logical next step to test whether they account for the observed clustering.

The third stage builds on the central theoretical argument of this paper, that spatial dimensions contribute to creating patterns of inequality [10,14], and includes theoretically relevant predictors based on the framework of Hooks et al., [12] that the interrelated factors of economic structures, institutional arrangements, and geographical context. The aim is to refine the model by adding variables representing municipality characteristics, thereby minimizing unexplained variance.

The fourth and final stage revisits the identification of hotspots based on residuals from the improved model. The purpose of this is to determine whether including spatial characteristics reduces unexplained variance to identify significant patterns of spatial clustering.

## 4. Results

### 4.1 Stage 1 – Initial model

Tables 1.1–1.4 in Table 1 display the results from the initial model, which shows expected outcomes for variables at the individual and family level. There is a clear effect of socioeconomic status on the likelihood of receiving measures from child welfare services. Young people from households with lower levels of income, occupational prestige, and education are significantly more likely to receive child welfare service measures. The effect of individual variables varies across age categories. In the two youngest age groups, the likelihood of receiving child welfare service measures increases with age, but this trend reverses from age 13 onwards, where younger individuals are more likely to receive measures. Young people with a Norwegian background are significantly more likely to receive measures in all age categories. Frequent moves between municipalities significantly increase the likelihood of receiving child welfare service measures for all age groups except the 0–5 group, with a similar effect observed for moves within municipalities in the 13–18 age group.

**Table 1:** The effect of individual, family and spatial characteristics on access to measures from child welfare services for different age groups.

	(1) Initial model	(2) Initial model	(3) Initial model	(4) Initial model	(5) Revisited model	(6) Revisited model	(7) Revisited model	(8) Revisited model
VARIABLES	0-5 years	6-12 years	13-18 years	19-22 years	0-5 years	6-12 years	13-18 years	19-22 years
*Individual level*								
Gender	1.067	0.912	1.052	0.784	1.158	0.780	0.914	0.613
(male = 0, female = 1)	(0.100))	(0.057)	(0.049)	(0.120)	(0.372)	(0.144)	(0.138)	(0.275)
Age	1.336***	1.060***	0.767***	0.618***		1.055***	0.780***	0.627***
	(0.045)	(0.012)	(0.012)	(0.035)		(0.014)	(0.014)	(0.038)
Country background	0.746***	0.867**	1.135**	0.648***	0.815	0.878	1.131**	0.732**
(Not Norway = 0, Norway = 1)	(0.088)	(0.067)	(0.072)	(0.117)	(0.127)	(0.078)	(0.081)	(0.155)
Within municipality moves	0.994	1.012	1.036***	1.031	1.032	1.022	1.034***	1.036
	(0.021)	(0.015)	(0.012)	(0.031)	(0.027)	(0.017)	(0.013)	(0.035)
Between municipality moves	1.036	1.063***	1.042***	1.128***	0.979	1.060**	1.038**	1.096
	(0.033)	(0.023)	(0.018)	(0.056)	(0.039)	(0.026)	(0.020)	(0.063)
*Family level*								
Income	0.801***	0.897***	0.925**	0.880	0.921	0.905***	0.942	0.899
	(0.425)	(0.032)	(0.033)	(0.073)	(0.070)	(0.036)	(0.039)	(0.084)
Occupational prestige	0.906***	0.935***	0.991	0.942**	0.884***	0.940***	0.993	0.940
	(0.021)	(0.014)	(0.014)	(0.033)	(0.027)	(0.016)	(0.015)	(0.037)
Education	0.876***	0.856***	0.921***	0.885**	0.853***	0.859***	0.913***	0.895
	(0.041)	(0.026)	(0.020)	(0.070)	(0.050)	(0.029)	(0.022)	(0.064)
*Municipality level*								
Semi-urban municipality					1.255	0.846**	0.991	0.705*
					(0.198)	(0.077)	(0.068)	(0.140)
Rural municipality					1.537***	0.968	0.987	0.636*
					(0.249)	(0.093)	(0.072)	(0.151)
Unemployment in municipalty					0.922	0.953	1.034	0.990
in %					(0.057)	(0.033)	(0.036)	(0.097)
Life expectancy					0.949	1.026	1.034	1.076
					(0.069)	(0.043)	(0.035)	(0.120)
Public housing in municpality						1.006	0.999	1.003
in %						(0.005)	(0.004)	(0.012)
Constant	2.123	1.535	32.123***	4551.959***	112.513	0.188	1.522	8.471
	(1.436)	(0.667)	(15.192)	(6686.539)	(669.234)	(0.663)	(4.328)	(78.857)
Observations	7,658	17,295	19,379	15,614	2,105	13,657	15,413	12,769

Standard errors in parentheses *** p < 0.01, ** p < 0.05, * p < 0.1.

**Note**: Table 1 displays data presented in odds ratio and with cluster-correlated standard errors.

### 4.2 Stage 2 – Mapping residuals

**Fig 1:**
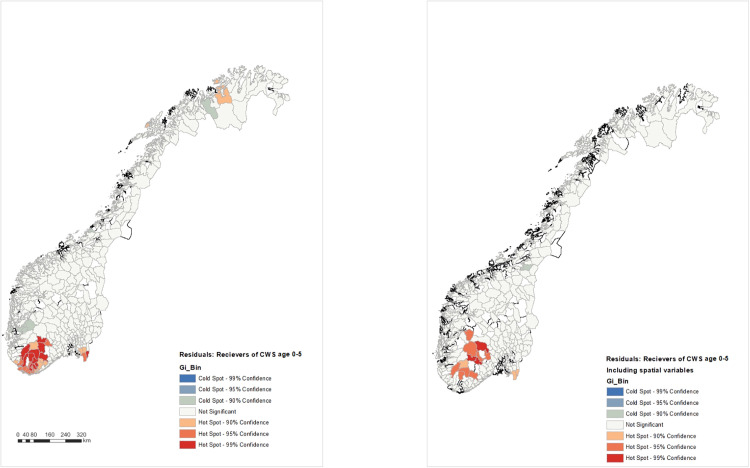
Hotspots and coldspots in distribution of child welfare services for different age groups without and with spatial variables.

Based on the initial model, the average residuals for each age group are calculated for each Norwegian municipality. This allows for mapping the spatial distribution of model residuals using the hotspot analysis function in GIS. Fig 1 contains four pairs of maps that illustrate this distribution. While there are differences in distribution across age groups, clusters of hotspots are consistently found in the western and southern parts of Norway, regardless of age group. The distribution of cold spots varies more across age groups, with clear cold spots in the northern Trøndelag for the 13–18 group and in various parts of Finnmark for the 0–6, 6–12, and 13–18 age groups. These findings suggest that the initial model alone cannot fully explain the provision of child welfare services in Norway, indicating the need for further model development.

### 4.3 Stage 3 – Revisiting the model

In this stage, the initial model is expanded to include spatial characteristics at the municipality level, as displayed in Tables 1.5–1.8. Logistic regression is performed for each age group, and the results indicate that the overall trends are consistent with the initial model, although there are some variations in significance levels for some age groups for variables such as country background, moving between municipalities, occupational prestige, income, and education. It should be noted that public housing data for the 0–5 age group are not included due to data limitations. As a result, direct comparisons between models should be approached with caution. Nevertheless, the overall trends remain meaningful and provide valuable insight for interpretation.

Few of the newly incorporated variables measuring spatial characteristics are significant. Living in a semi-urban municipality significantly decreases the likelihood of receiving child welfare service measures for the 6–12 and 19–22 age groups. Further, it is worth noting that living in a rural municipality significantly increases the likelihood of receiving measures for the youngest age group, while it significantly decreases the likelihood of receiving measures for the 19–22 age group. The other indicators for spatial characteristics show no significant effect on service provision. The Bayesian Information Criterion (BIC) indicates that the revisited model is a clear improvement over the initial model. As BIC tends to favor simpler models, this suggests that the extended model provides a better fit [53].

### 4.4 Stage 4 – Revisiting mapping residuals

New residuals for each age group are calculated based on the improved model, and the hotspot analysis function is used again to map clusters of high or low residuals. The inclusion of spatial variables has decreased the number of hotspots and cold spots, indicating less unexplained variance in the improved model. However, clusters of hotspots remain in the western and southern parts of Norway for all age categories, suggesting that the model is less effective in explaining the distribution of child welfare services in these areas. Notably, a cluster of hotspots appears for the 19–22 age group around Bodø in northern Norway. Compared to the initial model, the improved model results in fewer cold spots, although clusters of cold spots persist, particularly for the 6–12 age group in less central areas of eastern Norway. This suggests that there is still unexplained variance in the model, and such differences should be explored further, either by developing the model further or by using the areas with significant hotspots as a basis for future field work.

## 5. Discussion

The findings from this study emphasize that inequalities in child welfare services should be examined using a multidimensional approach, including spatial and temporal perspectives, thus supporting arguments made by researchers like Webb and Bywaters [8], Hooks et al. [12], and Harvey [10]. Including spatial characteristics, reflecting economic structures, institutional arrangements, and geographic context, reduces unexplained variance in the distribution of child welfare services, highlighting that the location and characteristics of these locations matter for access to services. Furthermore, access to services varies across different age groups, making temporal perspectives necessary in order to understand patterns of service provision.

This study highlights that access to child welfare service measures varies across different areas in Norway, supporting Hooks et al.’s, [12] argument that places are both markers and makers of inequality. The geographic context – represented here by level of urbanization – suggests that living in a semi-urban or rural municipality is associated with a lower likelihood of receiving child welfare measures for children aged 6–12 and for young adults aged 19–22. This aligns with the idea that spatial characteristics, such as remoteness and population density, shape institutional capacity and service accessibility [12,19].

According to Massey [13] and Harvey [10], underlying economic structures may shape patterns of service provision. Rural areas often rely on traditional industries with lower income levels and educational requirements [15], which can limit local tax revenues and reduce investment in public services. These structural conditions may explain the observed inequalities in service provision across age groups, where the youngest children are more likely to receive services in rural areas than older young people. Drange et al. [51] suggest that this may be due to transparency in rural municipalities, where concerns are more visible, and interventions occur earlier. This pattern may also reflect national policy priorities that emphasize early intervention as a strategy to prevent long-term disadvantage [32,33]. Paulsen et al. [5] further note that limited resources often lead services to prioritize younger children, driven by both perceived urgency and political encouragement [32,33]. Smaller municipalities often face challenges in maintaining specialized services due to limited staff, fewer routines, and greater vulnerability to staff turnover. The physical distance to other municipalities further complicates collaboration and knowledge exchange, forcing services to make strategic decisions about resource allocation. These constraints, combined with national priorities, result in a focus on acute cases. In other words, a combination of geographic context, economic structures, and institutional arrangements help explain observed discrepancies in the provision of child welfare services between places and age groups.

The findings of disparities in service provision raise important questions about equality in well-being. The lower likelihood of older young people in rural areas receiving support may have long-term implications, particularly given the multidimensional nature of well-being, which includes health, security, and quality of life. Inequality in access to services is also concerning for Norwegian society as a whole, given the state’s explicit goals of maintaining settlement patterns. Already, there is a trend of urban areas growing, while rural areas experience population decline and aging [54]. When rural areas struggle to provide equitable services, it may contribute to an increased urbanization, as families and young people may relocate to urban areas in search of better services and opportunities. This may undermine efforts to maintain settlement patterns and increase pressure on urban areas in areas such as service provision, housing, and the labor market. Consequently, addressing spatial inequalities in child welfare services is not only a matter of individual well-being, but also of sustainable regional development. Furthermore, the principle of the generalist authority system mandates that all municipalities should provide the same quality of public services [11]. Challenges with the provision and quality of public services in rural areas were also highlighted in an investigation by the Ministry of Local Government and Regional Development [11]. The report suggests that larger municipalities could help equalize differences between areas. Additionally, it emphasizes the importance of inter-municipal cooperation to create networks of competence, exchange experiences, and share resources. This paper attempted to explore the effect of inter-municipal cooperation on the provision of child welfare services, but no significant results were found and are therefore not explained further. However, the relationship between service provision and inter-municipal cooperation warrants further examination.

Revisiting the hotspot analysis after including spatial variables in the regression models revealed a decrease in the number of significant hotspots. This suggests that spatial variables contribute to explaining the geographic distribution of child welfare service measures and help reduce some of the unexplained variance. However, the persistence of significant hotspots – particularly in the western and southern regions of Norway – indicates that important explanatory factors remain unaccounted for in the current models. While the models include key variables related to economic, institutional, and geographic factors, they may not fully capture more nuanced or context-specific influences on service provision. For instance, factors such as local governance practices and priorities, or norms in the local society, may play a role but can be difficult to quantify in large-scale statistical models. Additionally, cultural or religious norms, which are known to vary regionally, could influence service provision. Uncovering such dynamics requires careful, context-sensitive analysis. To better understand the remaining unexplained variance, future research could expand the model with additional variables based on theory and empirical knowledge. Another approach is to use hotspot analysis as a foundation for qualitative case studies in selected municipalities. This would allow for a deeper exploration of local practices, cultures, and community dynamics that are not easily captured through quantitative data alone.

The findings from this paper emphasize the importance of including temporal perspectives when exploring inequality, aligning with the views of Gilligan and Brady [20] and Berg [9]. Temporal perspectives are valuable, as individual, family, and spatial variables have different effects at different stages in life. For instance, living in rural areas increases the likelihood of receiving services for the youngest age group, while the effect is negative for older young people. Additionally, young people below the age of 12 are more likely to receive measures from child welfare services than those older than 12. Although it is outside the scope of this paper to explore whether this reflects the actual needs of young people or is a result of policy and practice, it underscores the necessity of temporal perspectives in social services research.

Furthermore, the significant impact of family socioeconomic status highlights the need for temporal perspectives, as intergenerational effects strongly influence whether young people receive child welfare service measures. These findings have two potential implications. On one hand, they may indicate that the most vulnerable families are receiving support, suggesting that child welfare services help mitigate the reproduction of disadvantage. On the other hand, they could point to a selection bias in child welfare service recruitment, thereby perpetuating disadvantages over time. It is likely that the answer lies somewhere in between, necessitating further examination of these relationships. Consequently, applying temporal perspectives to explore inequalities in child welfare services highlights that young people are not a homogeneous group and should not be treated as such.

Findings from this paper, supported by previous research, emphasize significant geographical and temporal disparities in the provision of child welfare services in Norway. These disparities conflict with national policies that promote equal access to high-quality services regardless of location, as outlined in the generalist authority system [11]. This inconsistency is concerning, particularly when considering the goal of ensuring the well-being of all children. To address these inequalities, it is essential to identify where and how they occur. Recognizing patterns of disparity allows for the development of targeted interventions. Evidence from this and other studies, suggests that rural areas are particularly vulnerable and should be prioritized in policy responses. One approach is to adjust government funding mechanisms to promote equal service standards. This could involve reallocating resources through government incentives such as The General-Purpose Grant Scheme or Regional Policy Grants [55]. Another strategy is to strengthen inter-municipal cooperation. By sharing resources and expertise across municipalities, services can become more resilient and better equipped to reduce spatial inequalities. Although an evaluation of inter-municipal cooperation in Norwegian child welfare services does not conclude on the effect of such cooperation, and further examination is therefore necessary [24].

## 6. Conclusion

This study investigates how spatial and temporal dimensions influence the distribution of child welfare services in Norway. It builds on the understanding that places, through their social, economic, and institutional characteristics, shape and maintain patterns of inequality, alongside more traditional approaches focused on individual and family circumstances. The most significant finding is the strong influence of geographic context, particularly the level of urbanization, on service provision. Rural areas provide significantly fewer child welfare services than their urban counterparts. However, a notable exception is observed among young people between the ages of 0 and 5. This may reflect difficult prioritization and lack of material and human resources. It also points to a temporal dimension of inequality, where access to services varies not only across space but also over time for young people.

A central takeaway is that spatial and temporal factors must be integrated into analyses of inequalities in child welfare services. These dimensions reveal inconsistencies with national policies that promote equal access to services regardless of location. Recognizing these discrepancies is crucial for developing robust policies and practices in public services to mitigate the increasing inequality in Norway and thereby enhance the well-being of a marginalized group.

While spatial inequality matters, future research is needed to identify which specific spatial factors affect service distribution. Reducing the unexplained variance observed in this study will require refining the spatial framework and employing diverse methodological approaches. Additionally, studying municipalities that have implemented targeted incentives, such as inter-municipal cooperation, could yield insights into strategies for mitigating inequality.
