# Correction: Who gets what, where: To what extent are inequalities in distribution of Norwegian child welfare service measures related to spatial and temporal variation

**DOI:** 10.1371/journal.pone.0341689

**Published:** 2026-01-22

**Authors:** Norunn Hornset

Fig 1 was uploaded incorrectly. Please see the correct Fig 1 here.

**Fig 1:**
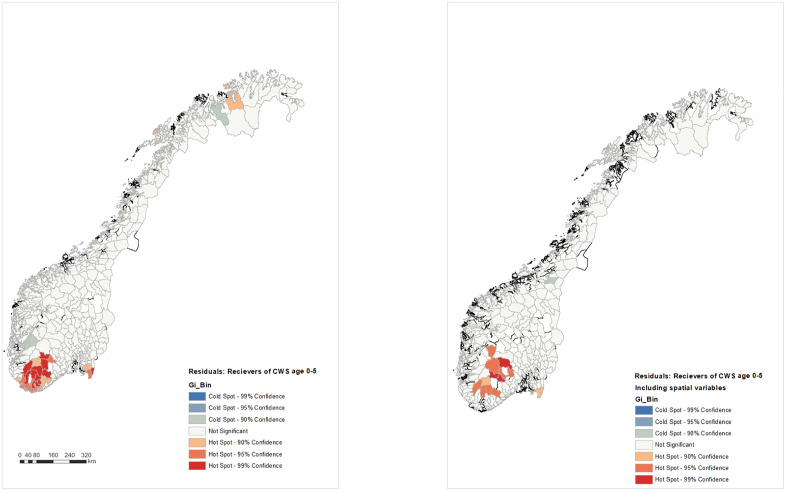
Hotspots and coldspots in distribution of child welfare services for different age groups without and with spatial variables.
